# Understanding the relationship between the perceived characteristics of clinical practice guidelines and their uptake: protocol for a realist review

**DOI:** 10.1186/1748-5908-6-69

**Published:** 2011-07-06

**Authors:** Monika Kastner, Elizabeth Estey, Laure Perrier, Ian D Graham, Jeremy Grimshaw, Sharon E Straus, Merrick Zwarenstein, Onil Bhattacharyya

**Affiliations:** 1 Li Ka Shing Knowledge Institute of St. Michael's Hospital, Toronto, Ontario, Canada; 2 Continuing Education and Professional Development, Faculty of Medicine, University of Toronto, Toronto, Ontario, Canada; 3 Department of Epidemiology and Community Medicine, Ottawa Health Research Institute, University of Ottawa, Ottawa, Ontario, Canada; 4 Faculty of Medicine, University of Toronto, Toronto, Ontario, Canada; 5 Sunnybrook Research Institute, Toronto, Ontario, Canada; 6 Family and Community Medicine, University of Toronto, Toronto, Ontario, Canada

## Abstract

**Background:**

Clinical practice guidelines have the potential to facilitate the implementation of evidence into practice, support clinical decision making, specify beneficial therapeutic approaches, and influence public policy. However, these potential benefits have not been consistently achieved. The limited impact of guidelines can be attributed to organisational constraints, the complexity of the guidelines, and the lack of usability testing or end-user involvement in their development. Implementability has been referred to as the perceived characteristics of guidelines that predict the relative ease of their implementation at the clinical level, but this concept is as yet poorly defined. The objective of our study is to identify guideline attributes that affect uptake in practice by considering evidence from four disciplines (medicine, psychology, management, human factors engineering) to determine the relationship between the perceived characteristics of recommendations and their uptake and to develop a framework of implementability.

**Methods:**

A realist-review approach to knowledge synthesis will be used to understand attributes of guidelines (*e.g*., its text and content) and how changing these elements might impact clinical practice and clinical decision making. It also allows for the exploration of 'what works for whom, in what circumstances, and in what respects'. The realist review will be structured according to Pawson's five practical steps in realist reviews: (1) clarifying the scope of the review, (2) determining the search strategy, (3) ensuring proper article selection and study quality assessment, (4) extracting and organising data, and (5) synthesising the evidence and drawing conclusions. Data will be synthesised according to a two-stage analysis: (1) we will extract and define all relevant guideline attributes from the different disciplines, then create a shortlist of unique attributes and investigate their relationships with uptake, and (2) we will compare and contrast the attributes and guideline uptake within each and between the four disciplines to create a robust framework of implementability.

**Discussion:**

Creating guidelines that are designed to maximise uptake may be a potentially effective and inexpensive way of increasing their impact. However, this is best achieved by a comprehensive framework to inform the design of guidelines drawing on a range of disciplines that study behaviour change. This study will use a customised realist-review approach to synthesising the literature to better understand and operationalise a complex and under-theorised concept.

## Background

Clinical practice guidelines are 'systematically developed statements to assist practitioner and patient decisions about appropriate health care for specific clinical circumstances' [1,2]. Guidelines have the potential to facilitate the implementation of evidence into practice, but these potential benefits have not been consistently achieved [3-5]. The limited impact of guidelines can be attributed to inconsistent adoption in clinical practice [2,6]. There are two general approaches to improving uptake of guidelines: (1) *extrinsic *implementation strategies, which target providers or practice environments to increase guideline adherence, and (2) *intrinsic *implementation strategies, which target guideline developers and end users to modify the guideline itself to facilitate adherence. A comprehensive review found that overall improvement in quality of care using extrinsic strategies was generally modest [7], and costs, when measured, were highly variable [7,8]. Intrinsic strategies that address attributes of guidelines involve the interaction between the guideline itself and the perceptions of its end user. The 'characteristics' of guidelines (such as design and wording) may be perceived differently by different people; for example, what is clear to one person may be confusing to another. We believe that if found to be effective, optimising characteristics of guidelines (as perceived by their end users) that are associated with uptake could be routinely incorporated into guideline development at minimal cost. Desirable attributes of guidelines, as described by the US Agency for Health Care Policy and Research, include validity, reproducibility, reliability, clinical applicability, clinical flexibility, and clarity [1,9]. Grol *et al*. found that guidelines that are compatible with existing norms among the target group and those that do not demand too much change to existing routines, extra resources, or acquisition of new knowledge and skills were used more [10]. Michie *et al*. suggest that clarity and specificity of behavioural instructions are important to get physicians to follow guidelines but have largely been overlooked [11]. Their work suggests that individuals are more successful at changing their behaviour if they have a more specific plan [12].

Shiffman *et al*. have referred to 'implementability' as the perceived characteristics of guidelines that predict the relative ease of their implementation [13]. Existing work on guideline implementability has been focused on the medical literature, but including disciplines focused on changing human behavior, such as psychology, marketing, and human-factors engineering, may provide deeper conceptualisation of its underpinnings, thereby improving the potential for better uptake of guidelines into clinical practice. Existing guideline tools assess the methodological quality of guidelines [14], rate the quality of evidence and strength of recommendations [15], inform developers about potential problems with implementation [13], and help adapt existing guidelines into other settings [16]. Components of these tools might contribute to successful implementation, but most do not fully consider end-user needs, are not informed by an explicit review of the relevant literature, and do not completely operationalise the concept of guideline implementability.

To better understand the concept of implementability and the relationship between characteristics of guidelines and their uptake by physicians, the objectives of our study are to answer the following questions:

1. What works, for whom, in what circumstances in relation to implementing guidelines?

2. What perceived characteristics of guidelines affect uptake of evidence-based recommendations in four disciplines: medicine, psychology, management, and human-factors engineering?

3. What is the relationship between the perceived characteristics of recommendations and their uptake by clinicians?

4. Which perceived characteristics of recommendations are most closely associated with uptake?

5. How are these perceived characteristics represented in the context of each of the four disciplines?

## Methods

The selection of our study methods was guided explicitly by our research questions. To select the most appropriate synthesis method, we assessed 10 potentially relevant review methodologies [17-21] and classified their features as being idealist or realist. Of the 10 synthesis methods, we identified the realist review [22], meta-narrative synthesis [23], and meta-ethnography [24] as the most potentially relevant for answering our research questions. We interrogated each of these three methods to decide which would be the most appropriate to use as our primary synthesis method in the context of our research questions.

Realist reviews provide a structured approach to a 'complete' review, including sampling, study quality assessment, data extraction, and analysis. They are helpful for interrogating underlying theories and mechanisms of implementability (*i.e*., how the attributes of guidelines affect uptake) and encourage the inclusion of quantitative and qualitative evidence. However, realist reviews lack a comprehensive process to compare disciplinary perspectives on a given issue. Meta-narrative synthesis is helpful for analysing data across different fields, constructing the narrative within a discipline, and comparing them between disciplines. However, it may not be able to interpret the specific intrinsic attributes of guidelines and their relationship with uptake. Meta-ethnography offers a systematic approach to synthesis to better understand specific attributes of guidelines and their relationship with uptake, but it considers only qualitative studies for inclusion and is a means of analysis only, offering little guidance on the complete process for conducting a review. Since none of the review methods are a 'perfect' fit, we will adopt a more flexible approach to reviewing the literature. We will select the realist review as our primary review method because it has the most potential for answering the majority of our research questions, is a structured and relatively transparent approach to conducting the review, and allows for the inclusion of both quantitative and qualitative evidence. During the analysis phase of the review, we will use realist-review analysis methods, but will also incorporate qualitative analysis techniques borrowed from meta-ethnography to translate definitions of guideline attributes between disciplines, condense them into a comprehensive set of unique attributes, and describe the relationships among them.

### Realist-review methodology

Pioneered by Ray Pawson, the realist review is an explicitly theory-driven approach to the synthesis of evidence-it seeks to interrogate the theories that underpin the programs or interventions being studied [22]. A realist synthesis takes a 'generative' approach to causation, that is, 'to infer a causal outcome (O) between two events (X and Y), one needs to understand the underlying mechanism (M) that connects them and the context (C) in which the relationship occurs' [25]. Its primary focus is to test the causal mechanisms or 'theories of change' behind interventions or programmes. In the context of guideline implementability, a realist review can thus facilitate the careful examination and understanding of the attributes of guidelines (*e.g*., its text, content, and presentation) and how changing these attributes might impact clinical decision making for physicians. A further benefit of the realist-review approach is that it seeks to explore 'what works for whom, in what circumstances, and in what respects' [22]. Other strengths of this approach are that it engages stakeholders throughout the review process and encourages the inclusion of diverse types of evidence (*i.e*., quantitative and qualitative) so that the processes and impacts of interventions can be investigated [22]. The current study will use five steps adapted from Pawson's practical steps in realist reviews [22,26].

### Step 1: clarifying the scope of the review

In a realist review, the inquiry is targeted to answering why, when, and how an intervention may or may not succeed [22,26]. In the context of guideline implementability, it will aim to build explanations across interventions that share similar underlying theories of change about why practice guidelines are not implemented successfully or why they do or do not facilitate knowledge uptake, for whom, in what circumstances, and how. This method is different from traditional systematic reviews, where the general approach to determining the research question(s) is to inquire simply whether a particular intervention works. The two approaches are nonoverlapping-realist reviews cannot answer whether something works, and quantitative systematic reviews will almost never have sufficient trials to answer how and why something works.

We will use several strategies to refine the purpose of the review. Using a theory-integrity strategy (*i.e*., does the intervention work as predicted), we will attempt to reveal the 'typical weak points and stumbling blocks in the history of the intervention' (in our work, the intervention will be defined as clinical practice guidelines) [22]. We will also try to uncover evidence to adjudicate between rival theories for uptake of guideline recommendations and to identify which alternate mechanism is most successful. Additionally, an important strategy will be to perform an exercise to determine for whom and in what circumstances guidelines are implemented successfully. This will be done by uncovering studies of the same strategies for guideline uptake but in different settings to identify the 'winners and losers'. This will clarify our understanding of why certain strategies work only under certain circumstances and for only certain populations [22], and may also reveal which attributes of guidelines influence their uptake.

### Key theories to be explored

Prior to conducting the review, the body of working theories that 'lies behind the intervention' needs to be identified. Pawson suggests tapping into stakeholders and experts as an initial strategy to help frame the problem [22,26]. Our approach to exploring key theories will begin by consulting with clinician scientists and experts in guideline development and knowledge translation to better understand perceptions of guideline implementability before searching the literature to identify 'theories, hunches, expectations, and the rationalizations' for why they may or may not facilitate knowledge uptake [22,25]. The goal of this exercise is not to collect data about the efficacy of guidelines but to identify a range of theories and explanations for how guidelines are supposed to work (and for whom), when they do work, when they don't achieve the desired change in practices, why they are not effective in this, and why they are not being used. The body of literature from exploring key theories will be representative of our first stage of literature searching (*i.e*., the core articles search as described below), from which we will build a working list of candidate theories (*i.e*., middle-range or 'educated guess' theories). These candidate theories will be continuously tested and appended as they evolve (or new theories emerge) and will be finalised only when their validity has been tested and explored during the realist-review process [22,27].

Well-studied theories related to changing behaviour include the Social Cognitive Theory [28], the Theory of Reasoned Action [29], the Theory of Planned Behaviour (TPB) [30], the health belief model [31], stages of readiness to change [32], and Rogers' Diffusion of Innovations Theory [33]. To guide our exploration of which perceived factors influence guideline adherence, we will use the TPB, as it is the most widely researched, influential, and empirically based framework designed to predict and explain human behaviour in specific contexts [30,34]. According to the TPB, human behaviour is guided by three types of motivational factors that can lead to intention to perform the target behaviour: (1) attitudes toward the behavior, (2) subjective norms (*i.e*., a person's perception of injunctive norms [behaviours perceived as being approved by other people] and descriptive norms [people's perception of what is commonly done in specific situations]), and (3) perceived behavioural control [30]. In the context of guideline implementability, the central behavioural goal is to 'use' or 'uptake' guidelines. These intentions can be illustrated according to the three conceptually independent predictor variables. The first can be conceptualised as the attitude or behavioural beliefs toward using guidelines and refers to the degree to which a person has a favourable or unfavourable evaluation of this behavioural goal (*i.e*., the strength of their intention or motivation). The second predictor is normative beliefs (*i.e*., the subjective norm), which refers to the perceived social pressure to use or not use guidelines. The third predictor is the degree of perceived behavioural control, which can be conceptualised as the perceived ease or difficulty of performing guideline use or uptake. This may reflect past experiences as well as anticipated impediments and obstacles of the behaviour. Together, these three predictor factors can lead to the formation of behavioural intention. In general, we can predict that the more favourable the attitude and subjective norm with respect to using guidelines, and the greater the perceived control, the stronger the individual's intention to adhere to them. Intention is thus an immediate antecedent of guideline use, but the degree of success will also depend on other nonmotivational factors, such as availability of requisite opportunities and resources (*e.g*., time, resources, skills, willpower) [30]. Based on the TPB, it is expected that intentions to use/uptake guidelines will be predicted from attitudes, subjective norms, and perceived control with respect to this goal and that intentions and perceived control may in turn permit prediction of actual adherence to guidelines.

### Preliminary list of candidate theories

Our preliminary list of candidate theories are as follows:

1. Clinical practice guidelines are not used by physicians in part because of specific perceived guideline characteristics (*i.e*., attributes of implementability). For example, guidelines and their recommendations are too complex, lengthy, and time consuming to use and are difficult to follow (*e.g*., ambiguous language)

2. There are 'trade-offs' between various guideline attributes that hinder or facilitate uptake (the examination of the trade-offs between the various dimensions will help clarify our understanding of how and why this theory makes sense).

### Step 2: determining the search strategy

There are two key differences in searching between realist reviews and traditional systematic reviews. In realist reviews, there is no finite set of relevant articles that can be defined and then found. In contrast, traditional systematic reviews often take a linear, time-restricted approach to searching the literature, striving for completeness by attempting to identify every single paper on a given topic or intervention [22,35]. The second difference is that primary studies in realist reviews are rarely the unit of analysis, so studies are not excluded based on rigour, as this would reduce rather than increase the validity and generalisability of the findings. Instead, it is the relevant elements of the primary study that are tested for specific hypotheses about the link between context, mechanism, and outcome [22]. We initially attempted a traditional search with text words and MeSH terms (identified from the preliminary list of 20 relevant core articles) in MEDLINE using an Ovid (Ovid Technologies, Inc., New York, NY, USA) interface to verify whether this strategy would have merit for capturing other potentially relevant articles. Of the over 5,000 articles that were generated, only 8 of the 20 relevant core articles were identified (40%), indicating that this strategy would likely be inefficient and resource intensive (*e.g*., duplicate reviewing from a large search retrieval with a low potential for identifying relevant articles). This finding is consistent with Greenhalgh *et al*.'s review of complex evidence (the diffusion of service-level innovations in healthcare organisations) [36], which found that protocol-driven search strategies performed poorly when identifying potentially relevant articles for systematic reviews of complex evidence-only 30% of their sources were identified through protocol-driven strategies (*i.e*., electronic database and hand searching), whereas snowball sampling (*i.e*., reference and citation tracking) yielded the majority of relevant articles (51%) [37]. In fact, recent work has shown that asking experts where to look for potentially relevant articles is an effective strategy [37,38]. We will thus use snowball sampling to identify experts in the four discipline areas, who will then be consulted to direct us where to look for and identify potentially relevant literature and concepts.

**Figure 1:**
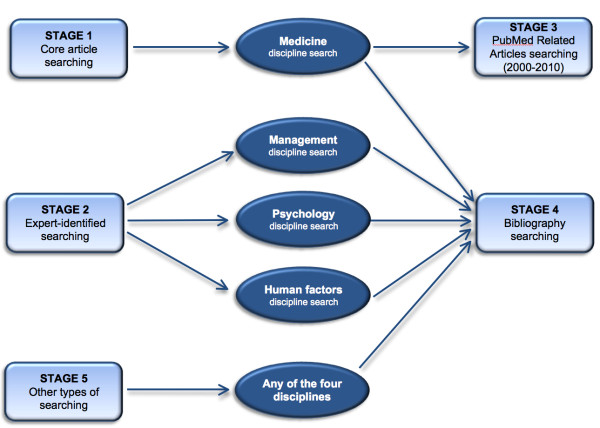
**Search schematic of the Realist Review**.

We will use the multiple-search strategy approach of realist reviews, which seeks to explore and contextualise the intervention in multiple settings. This will thus be an iterative, interactive, and purposive sampling strategy with no predefined sampling frame [26,35]. Searching will resemble the sampling strategies of qualitative research: purposive, snowball (*i.e*., manually searching for references of references or the process of identifying cases from people who know people who have relevant information), or opportunistic sampling for information-rich cases, with the goal of retrieving materials to answer specific questions or to test particular theories. This process requires taking a more flexible and iterative approach to the literature to capitalise on unanticipated findings. We will also consider a model of searching called 'berrypicking', which asserts that typical search queries are not static but evolve, gather information in 'bits and pieces rather than in one grand best retrieved set', and use a wide variety of search techniques and sources beyond common bibliographic databases such as MEDLINE [39]. Our strategy will thus consist of five nonlinear and iterative stages of searching (see Figure 1 for the algorithm of this process), as outlined below.

### Stage 1: background search for core articles

The purpose of the background search is to 'get a feel for the literature' to determine what and how much information exists, in what form, and where it is. We gathered a preliminary core set of articles using a 'desk drawer' search strategy (*i.e*., going through existing materials of the research team). We then conducted a scoping review in MEDLINE and EMBASE using the following initial list of search terms, which were compiled from the collective knowledge of our research team consisting of clinician scientists and knowledge-translation researchers: 'implementability/implementation', 'clinical practice guidelines', 'knowledge translation'.

### Stage 2: expert-identified searching from multiple disciplines

To gather the comprehensive evidence needed, our strategy will involve searching the literature across four different disciplines relevant to the topic (*i.e*., medicine, psychology, management, and human-factors engineering), as we believe this will provide a broader insight into the concept of implementability. Snowball sampling will be used to identify experts in the four discipline areas, who will then be consulted to direct us where to look for and identify potentially relevant literature and concepts. This may also involve purposively searching discipline-specific databases for articles suggested by key experts in the four discipline areas.

### Stage 3: PubMed related-articles searching

We will search for additional articles by utilising the Related Articles feature in PubMed for articles retrieved from the various search stages and those deemed highly relevant by the core research team (limited to articles published between 2000 and 2010). This strategy was selected because previous work to identify optimal approaches to updating systematic reviews [40] or to verify that potentially relevant articles were not missed in a systematic review [41] has shown that the Related Articles feature in PubMed can identify most new 'signaling evidence' with a relatively low screening burden of new records per review [40,41].

### Stage 4: bibliographic searching of relevant articles

We will look for other potentially relevant articles using snowball sampling (*i.e*., scanning the reference lists of relevant articles) from stage 1 (core articles) and stage 2 (expert directed) searching.

### Stage 5: other types of searching

We will look for other potentially relevant articles by purposively scanning the literature of key authors and the articles discovered through snowball and opportunistic searching and serendipitous discovery. This stage will also include searching for grey literature: (a) websites, such as those for the Agency for Healthcare Research and Quality, Institute of Medicine, and various foundations (*e.g*., Robert Wood Johnson Foundation), and (b) approaching each discipline expert to identify unpublished documents (*e.g*., the Guideline Implementation Network and the National Guideline Clearinghouse [Expert Commentaries, AGREE {Appraisal of Guideline Research and Evaluation} Collaboration]).

### Deciding when to stop searching

Setting a threshold for stopping the search is an important consideration for conducting systematic reviews. In realist reviews, searching continues in a cyclical and iterative process that is not designed to be exhaustive. However, it remains important to not only create parameters to decide which studies are 'fit' for identifying, testing, or refining the theories but also to decide when a sufficient amount of evidence has been assembled to satisfy the theoretical need (*i.e*., to reach theoretical saturation) or to answer the research questions [22,35]. Pawson suggests that the 'test of saturation' can be applied iteratively, by asking after each stage or cycle of searching whether the latest sample of literature has added anything new to our understanding of the intervention and whether further searching is likely to add new knowledge [22,35]. As such, it is not possible to state the stopping point of searching *a priori *or to determine the number of studies at which theoretical saturation will occur. However, the reporting of this process will be transparent, and each step will be carefully documented.

### Step 3: article selection and study quality assessment

Although realist reviews acknowledge the principle that a quality filter should be applied at some point during the evidence synthesis, Pawson rejects the 'hierarchy of evidence' approach to study quality assessment [22]. He argues that multiple methods are needed to 'illuminate the richest picture' [22]. This involves testing for relevance (*i.e*., does the research address the theory under investigation, why guidelines are not implemented and in what context this occurs?) and rigour (*i.e*., does the research support the conclusions drawn from it?) [22]. Two reviewers will independently select articles (during title/abstract and full-text review) using a preliminary set of inclusion/exclusion criteria (which will evolve during the process of the review) (see Additional file 1). The purpose of the duplicate article-review process is partly to ensure a certain level of rigour (*i.e*., to correctly interpret the inclusion/exclusion criteria because we anticipate a steep learning curve). We also anticipate that the duplicate review process will serve as a great platform for reflexive discussion that will enable informed decisions among reviewers for identifying relevant data [38]. If there is strong agreement, it would possibly reduce the number of articles that would need to be reviewed in duplicate, given that we anticipate a high volume of potentially relevant articles. Inclusion criteria are articles that provide information about guideline attributes, address any aspect of why guidelines are not implemented for intrinsic reasons, and include perceptions of guideline developers or end users (*e.g*., physician providers) about intrinsic factors that influence intentions to use guidelines. We will define *guidelines *in other disciplines as any recommendations or guidance for behaviours that are consistent with those of clinical practice guidelines in medicine (and *implementability *will be defined as the uptake of recommendations). For example, guidelines might include instructions for mortgages or financial statements (management) and technical manuals for products (human-factors engineering). Exclusion criteria are opinion-driven studies (*i.e*., editorial reviews, commentaries, and letters), non-English language articles, articles that focus on how guidelines were developed or do not discuss the reasons for why guidelines are not implemented, and articles that discuss guideline implementation strategies that are extrinsic.

The process for determining 'rigour' is described by Pawson in terms of 'whether a particular inference drawn by the authors has sufficient weight to make a methodologically credible contribution to the test of a theory' and to apply 'judgment' to supplement formal critical appraisal checklists (if they are used) [22]. Applying judgment cannot be translated into a technical procedure, which is likely the reason why it has not been described in detail in published examples of realist reviews [27,42]. Our strategy will be to use rigour as a mediating tool rather than a testing method for article selection so that we can determine which studies best fit our purpose (*e.g*., for studies that have the same concepts but with differing methodological rigour or to adjudicate between studies that have similar methodologies but conflicting results). We will apply judgment to resolve conflicts amongst reviewers by considering whether the results can be applied to the context of healthcare providers using clinical practice guidelines. We want to be careful not to exclude articles based on methodological rigour alone, as the primary studies contribute different elements to the rich picture that constitutes the overall synthesis of evidence. In realist reviews, the study itself is rarely ever used as the unit of analysis; instead, realist reviews may consider small sections of the primary study (*e.g*., the *Introduction *or *Discussion *sections) to test a very specific hypothesis about the relationships between context, mechanism, and outcomes [22]. We will thus select and review studies based on what new knowledge they bring to our thinking about the theory of implementability. The meaning and value of rigour will then be defined, examined, and documented for each article. For example, we will document whether the source of an explanation for why guidelines are not implemented is supported by evidence or author opinion within the article. We will then use this information to mediate between studies of variable quality but with comparable relevance. The importance of transparency in the realist-review process parallels systematic reviews, to ensure that findings and conclusions are valid, reliable, and verifiable [26,35].

### Step 4: extracting and organising data

Two researchers will independently extract data from all potentially relevant full-text articles using a standardised data collection form, including the article citation, at which level it was searched (*e.g*., stage 2 expert-identified searching), discipline (*e.g*., medicine, psychology), study design, relevance, and the name and author's definition and operationalisation of the guideline attribute that was discussed in the article (see example in Additional file 2). However, interpretation of this data will be guided by judgment of the reviewers.

### Step 5: synthesising the evidence and drawing conclusions

We will synthesise data using several analytic approaches. First, we will use the realist-review approach to interrogate our final theory, which will be to determine 'what is it about practice guidelines that works (*i.e*., to facilitate uptake), for whom, in what circumstances, in what respects, and why'. We will then borrow synthesis methods from meta-ethnography to identify and interrogate specific guideline attributes and their potential trade-offs as well as their relationship with uptake by physicians [24]. The process of analysis will thus follow a two-level analysis, where the data will get further dissected and refined with each level of analysis.

### Level 1: realist-review analysis [22,35]

We will first explore what have been the typical weak points and major stumbling blocks (*i.e*., the barriers and facilitators) of guideline implementation by family physicians. The logic behind this approach is that interventions are only as strong as their weakest link. We will then look for rival theories of implementability (if they exist) to refine the understanding of how practice guidelines work by using evidence to 'adjudicate' between these rival theories of implementability. Next, we will consider the same theory in different settings. This approach assumes that particular intervention theories may work in some settings but not in others. We will attempt to make sense of the patterns of data that relate to the facilitator and barrier circumstances in which guidelines are successfully implemented or not. Finally, we will attempt to synthesise the data by comparing official expectations with actual practice (*i.e*., the expectation that family physicians will use clinical practice guidelines even though evidence indicates otherwise). This approach is particularly useful for comparing the 'official' theory (*i.e*., what specific content in guidelines should be used in what circumstances and how) and what actually happens in practice.

### Stage 2: qualitative analytic techniques

Although the realist-review analysis technique is helpful for interrogating our underlying theory, it lacks the process for interpreting the specific attributes of guideline recommendations that may facilitate guideline uptake and the process for associating the relationships among these dimensions to better understand their anticipated trade-offs. For this purpose, we will use various qualitative analytic techniques, drawing from Noblit and Hare's meta-ethnography [24]: reciprocal translation analysis (RTA), which can be used for instances when the accounts in an article are similar; refutational analysis, which can be used when the accounts are contradictory and an attempt is made to explain them; and line of argument (LOA) analysis, which can be used when inferences can be made by building up a picture grounded in the findings of separate studies. These three methods will be used to generate a complete list of unique guideline attributes and their definitions and will represent both an integrative and interpretive approach to revealing the relationships between guideline attributes and uptake [18,24,43].

RTA and refutational methods will first be used to translate definitions of guideline attributes from different disciplines into one another (*i.e*., how a concept in one paper is included in interpretations offered by other papers) and then LOA analysis will be used to come up with second- or third-order interpretations. For example, themes can be compared across studies and matched from one study to another (using RTA), ensuring that a key theme captures similar themes from different studies. We will begin this process by creating a list of themes or metaphors related to guideline attributes and determining how they are related (*e.g*., we might end up with different terms or definitions for the same attribute or the same attribute with different terms or definitions). This integrative approach allows these terminologies to be combined so that the differences between attribute terms can be negotiated to decide which might be the most relevant in the context of medicine. The RTA can then proceed to higher-order interpretations using the LOA synthesis method. According to Schutz's notions of 'orders' of constructs [44], synthesis and interpretation of first- and second-order constructs can be further distilled to reveal a new model, theory, or understanding (*i.e*., third-order interpretation) [45]. For example, first-order interpretations may represent the general understanding of guideline attributes as it relates to implementability and second-order interpretations may represent the explanations and theories used by authors in primary study reports (*i.e*., how the study author understands the concept). It is possible to then build on and extend these interpretations to reveal third-order constructs, which represent a new model, theory, or understanding. For example, the way in which guideline implementability is understood in the four disciplines (second-order interpretation) may be distilled further to reveal their relevance in the context of medicine (third-order interpretation). The output from these analyses is called the 'synthesizing argument', which represents the integration of evidence across studies into a coherent theoretical framework (similar to the analysis that is done in primary qualitative research) [18]. RTA and LOA synthesis methods will thus enable the organisation of guideline attributes and their trade-offs and interpret them according to how their relationships can be mapped to reveal the implications of these trade-offs in clinical practice.

## Discussion

Implementation research is complex, given the interplay of patient-, provider-, organisation-, and system-level factors. This is likely why the impact of implementation strategies has been modest, and general conclusions about which strategy should be applied in what context have been so limited [44]. Our work will help explain the intrinsic reasons for why and under what circumstances guidelines are not being implemented. This will be an important first step toward better understanding which attributes of guidelines have the potential to improve uptake in clinical practice. Depending on the findings, we will attempt to organise the results into a conceptual framework of implementability and identify attributes that can feasibly be changed during the guideline-development process.

Our work also represents a novel approach to knowledge synthesis. We will test how the use of a customised approach to synthesising the literature can answer research questions around a complex and under-theorised concept such as guideline implementability. Although we initially considered conducting a systematic review, there is increasing evidence that this may not be the most appropriate method for investigating complex and multidisciplinary topics [37]. Analysis of opposing epistemologies helped short-list potentially relevant synthesis methodologies, but in the process of choosing the realist review as the primary synthesis method, we discovered that many underlying principles of other synthesis methods were highly applicable but insufficiently covered all our questions-we had to use a hybrid model as there was no perfect fit with any of the available methods. This highlights the need for a more flexible approach to conducting literature syntheses of complex evidence, which may require borrowing relevant components of existing synthesis methods in coordination with a primary synthesis method (including Cochrane-style reviews) to complete the review. There is a need to shift the way we think about and conduct reviews of complex interventions and recognise that traditional systematic reviews may not always be the most appropriate. We should approach answering synthesis questions the same way we do when deciding the most appropriate study design for a primary study-by matching the appropriate design to fit the question or considering a mixed-methods design to better understand the how and why of effectiveness findings. In our study, we will show that a realist-review-informed synthesis combined with analysis components of meta-narrative and meta-ethnography techniques can be an effective strategy for discovering the unique attributes of guidelines that affect uptake across the disciplines of medicine, psychology, management, and human-factors engineering.

## Competing interests

The authors declare that they have no competing interests.

## Authors' contributions

All authors participated in the design of the study. MK drafted the manuscript, and all authors reviewed and approved the final manuscript.

## Supplementary Material

Additional file 1 **Inclusion/exclusion criteria**. Click here for file

Additional file 2 **Example of the data extraction form**. Click here for file
