# Supplementary material for: Understanding the relationship between the perceived characteristics of clinical practice guidelines and their uptake: protocol for a realist review
Source: Implement Sci. 2011 Jul 6;6:69. doi: 10.1186/1748-5908-6-69 (PMC3224565; doi:10.1186/1748-5908-6-69)
Supplement: Additional file 1 — Inclusion/exclusion criteria. [file 1748-5908-6-69-S1.DOC]

**Additional file 1:** **Inclusion/exclusion criteria**

|  | **Inclusion criteria** | **Exclusion criteria** |
| --- | --- | --- |
| **Study design** | - - Theory or conceptual framework   - Systematic review   - Narrative or overview reviews   - Randomised controlled trial (RCT)   - Non-RCT experimental or quasi-experimental study   - Questionnaire survey   - Qualitative interview study (including focus group)   - Ethnographic study   - Mixed-methodology case study   - Action research   - Tool/checklist/model   - Guideline/protocol   - Comparative case study   - Network analysis   - Attribution study | - - **Opinion-driven studies** - Editorial review - Commentary (except for expert-informed commentary) - Opinion piece - Letters - **Books or book chapters** - **Non-English articles** |
| **Population** | - All | - None excluded |
| **Intervention** (*i.e.*, clinical practice guidelines) | - Implementability can be defined as uptake of recommendations in the psychology, management, and human-factors engineering literature - Guidelines are defined in other disciplines as the extent to which recommendations can influence/affect behaviour such as   - management: instructions (*e.g.*, for mortgages, financial statements);   - human factors: technical manuals (*e.g.*, for products); protocols, scripts (*e.g.*, emergency procedures);   - psychology: to be determined | - Not applicable |
|  | - Any article about *why* guidelines are not being used/followed/implemented—reason needs to be intrinsic (*i.e.*, about guideline itself) | - If article is about *how* guidelines were developed - If article is about *if* guidelines are not being used/followed/implemented |
|  | - Any intrinsic factor/attribute/dimension of guidelines - Intrinsic factor is defined as any characteristics of the guideline or recommendation itself that may influence guideline implementability (*e.g.*, wording, format)   - other definitions for ‘intrinsic’ will be identified during data abstraction | - Any extrinsic factors/dimensions/attributes of guidelines - Extrinsic factors are defined as behavioural or environmental factors that may influence guideline implementability (*e.g.*, motivation of the provider to use the guideline; practice setting)   - other definitions for ‘extrinsic’ factors will be identified during data abstraction |
|  | - Any article that includes the perceptions of guideline developers or end users (providers) about intrinsic factors that influence intentions to use guidelines (along with many non-guideline-related factors) | - If the perceptions are related to extrinsic factors |
