# Supplementary figures and images for: Understanding the relationship between the perceived characteristics of clinical practice guidelines and their uptake: protocol for a realist review
Source: Implement Sci. 2011 Jul 6;6:69. doi: 10.1186/1748-5908-6-69 (PMC3224565; doi:10.1186/1748-5908-6-69)

**Additional file 2: Example of the data extraction form**

**
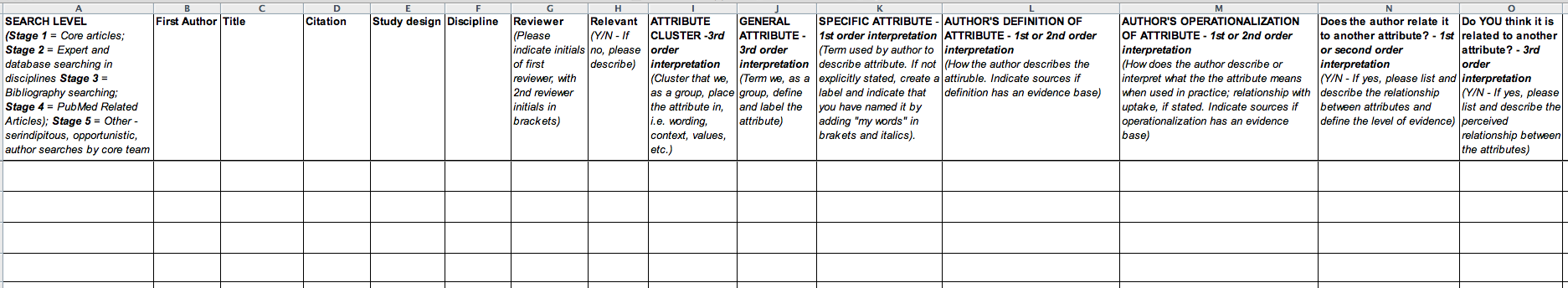
**

Supplement: Additional file 2 — Example of the data extraction form. [file 1748-5908-6-69-S2.DOC]
